# Associative memory cells and their working principle in the brain

**DOI:** 10.12688/f1000research.13665.1

**Published:** 2018-01-25

**Authors:** Jin-Hui Wang, Shan Cui

**Affiliations:** 1 College of Life Sciences, University of Chinese Academy of Sciences, Beijing, 100101, China; 2 School of Pharmacy, Qingdao University, Qingdao, Shandong, 266021, China; 3 Institute of Biophysics, Chinese Academy of Sciences, Beijing, 100101, China

**Keywords:** Associative memory cell, synapse, neuron, learning, cognition, brain

## Abstract

The acquisition, integration and storage of exogenous associated signals are termed as associative learning and memory. The consequences and processes of associative thinking and logical reasoning based on these stored exogenous signals can be memorized as endogenous signals, which are essential for decision making, intention, and planning. Associative memory cells recruited in these primary and secondary associative memories are presumably the foundation for the brain to fulfill cognition events and emotional reactions in life, though the plasticity of synaptic connectivity and neuronal activity has been believed to be involved in learning and memory. Current reports indicate that associative memory cells are recruited by their mutual synapse innervations among co-activated brain regions to fulfill the integration, storage and retrieval of associated signals. The activation of these associative memory cells initiates information recall in the mind, and the successful activation of their downstream neurons endorses memory presentations through behaviors and emotion reactions. In this review, we aim to draw a comprehensive diagram for associative memory cells, working principle and modulation, as well as propose their roles in cognition, emotion and behaviors.

Associative learning is an experience where multiple exogenous signals are jointly acquired through sensory systems. Associative memory involves the integration and storage of these associated signals in nerve cells, whose achievement can be proved by memory retrieval (recall and representation) via behaviors. Associative learning and memory is a common form of information storage for cognition throughout life ^1– 4^. In associative learning and memory, characteristic signals of each object or an environment are detected by distinct sensory modalities and cortices. These cross-modal signals are integrated for their associative storages. For instance, an orange is detected by the olfactory system for aromatic odor, the visual system for shape and color, the taste system for sweetness, the auditory system for name and so on. After associative memory forms, one of these signals induces the recall of other associated signals, and these signals are retrieved reciprocally, i.e., a signal induces the recall of its associated signals, or the other way around. How do sensory cortices integrate these signals, during initial associative learning, for associative storage, such that individuals can describe the object or environmental features? How are these associatively stored signals retrieved distinguishably in the brain? Although plasticity in the strength of synaptic connections and neuron activity is presumably a cellular mechanism for learning and memory ^5– 8^, the potentiation or depression of synaptic and neuronal activities in a pathway cannot account for the integrative storage of associated signals. Current reports show that nerve cells are reformed to be associative memory cells based on new synapse innervations from co-activated sensory cortices for the integrative storage of associated signals ^2, 3, 9– 15^.

In cognitions, logical reasoning, associative thinking and integrative imagination to these stored exogenous associated signals generate their secondary integrations, which can be stored as well as subsequently be recalled and represented. The storage of these secondary integrated signals (i.e., secondary associative memory) are essential for comparison, computation, decision making and planned intention under the consciousness condition ^3^. Although the memory occurs presumably in the prefrontal cortex, hippocampus, amygdala and so on ^16– 28^, whether these studies are involved in the second grade of memory based on information storage in primary associative memory cells of sensory cortices remains unknown ^3^. Current reports show that secondary associative memory cells are detected in the prefrontal cortex and the motor cortex, which are located at the downstream of primary associative memory cells ^29– 31^. 

To investigate the formation and working principle of associative memory cells, we need more complete animal models for associative memory, which can match the features of associative memory in human beings. Associative memory in humans comprises of a signal inducing the recall of its associated signals, or the other way around, for logical reasoning, associative thinking and imagination in forward and backward manners. In animals, fear conditioning, eyelid-blinking conditioning and operant conditioning are used to examine cellular and molecular mechanisms underlying associative memory ^32– 35^. After eyelid-blinking conditioning or fear conditioning based on sound signal is established, whether air-puffing to the cornea or electrical shock to the feet induces the recall of the sound signal remains unknown. In addition, the electrical shock may activate entire sensory cortices and even the whole brain by spreading electrical current in the body, so that the association is not region-specific in the brain. Current studies indicate that the associations of whisker and olfactory stimulations in mice lead to odorant-induced whisker motion and whisker-induced olfactory response ^2, 12– 14^, a comprehensive model to investigate associative memory. In this review, we aim to summarize associative memory cells and their working principles.

## Cellular changes in associative memory

Based on studies with animal models including fear conditioning, eyelid-blinking conditioning and operant conditioning in rodents ^32– 36^ and withdrawal reflex in Aplysia ^37– 39^, the following mechanisms may be involved in associative memory. From a psychological perspective, a conditioned signal induces the prediction of an unconditioned signal forthcoming as the basis of a conditioned reflex; however, the neural substrates remain unclear ^4^. In terms of memory location, the movement-related brain areas and motor neurons ^1, 40– 42^, or the sensory cortices ^43, 44^ presumably encode the storage of those associated signals; however, the characteristics and working principle of these neurons that are working in coordinated manner for memory formation remain to be elucidated ^2, 3^. In terms of the functional status of synapses and neurons, activity-dependent plasticity, e.g., long-term potentiation and depression of synaptic transmission ^45, 46^, is presumably involved. How these types of neuronal plasticity in a given pathway are correlated with the integration and storage of these associated signals has not been addressed. In addition, perceptual memory presumably resides in the cell assemblies formed by the strengthening of neuronal connections due to their correlated activities in information acquisition ^47^. However, the natures of these cell assemblies, the patterns of neuronal connection strengthening, and the correlation between cell assemblies and their strengthening are largely unknown.

Obviously, the current knowledge as indicated above does not inform us of the neural substrates for integrative storage of associated signals or their working principles. Neural plasticity alone cannot account for memory patterns, such as explicit versus implicit memory, declarative versus non-declarative memory, episodic versus semantic memory and transformation between such patterns ^48^, as well as the temporal features of memory from sensory inputs toward short-term and long-term memory. How memory constitutes the foundation of cognitive processes, such as associative thinking, logical reasoning and so on, remains unknown. How endogenous signals generated from logical reasoning and associative thinking is memorized for future presentation remains unknown. How memory is encoded under the different consciousness states is largely unclear. Thus, a comprehensive understanding of cellular mechanisms underlying associative memory should be established like an effort to see individual tress and the forests as well.

## Associative memory cells in sensory cortices

Associative memory cells are presumably recruited for the integrative storage of associated signals ^49^. The association of sensory signals leads to their integrative storage and retrieval, so that each signal evokes the recall of its associated signals. In terms of cellular substrate, the co-activation of sensory cortices induces mutual synapse innervations among these cortices, and recruits associative memory cells to integrate and encode these associated signals ^2, 13, 14^.

**Figure 1.**
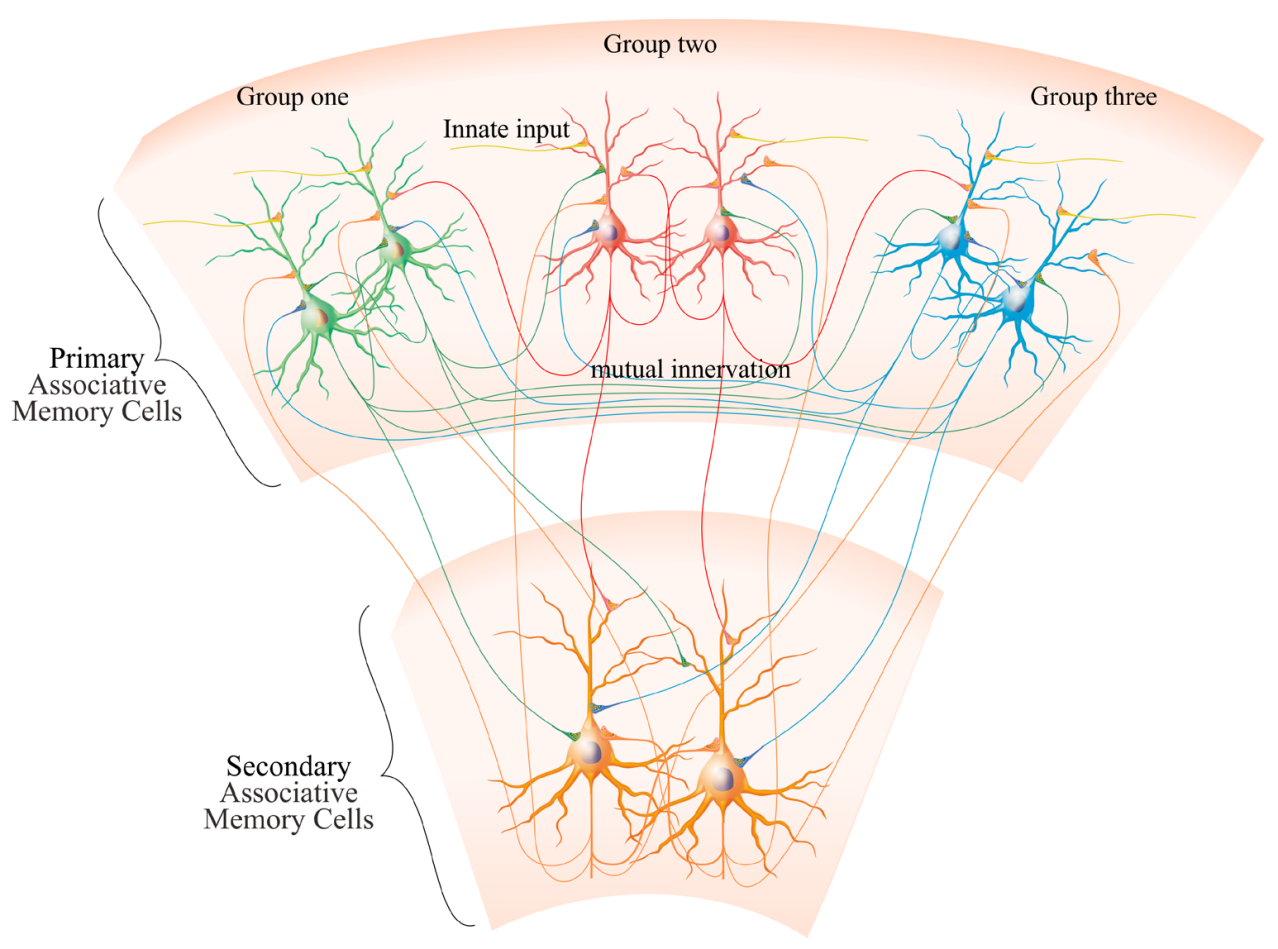
Associative memory cells and their connections. Top includes three groups of primary associative memory cells (green, red and blue) in the sensory cortices. There are mutual innervations among associative memory cells in each group (intramodal) and among three groups of associative memory cells (cross-modal). Bottom demonstrates secondary associative memory cells (orange) in cognition- and emotion-related brain area, and where these cells are mutually innervated. The axons of primary associative memory cells project to secondary associative memory cells whose axons project back to primary associative memory cells. All of neurons possess innate synapse innervations (yellow axons).

In the study of this assumption, associative learning by paring whisker and odor stimulations in mice leads to odorant-induced whisker motion and whisker-induced olfaction response. Their barrel cortical neurons are able to encode new odor signals and innate whisker signals, as well as receive new synapse innervations from the piriform cortex alongside innate ones from the thalamus. Their piriform cortical neurons encode new whisker signals and innate odor signals, as well as receive new synapse innervations from the barrel cortex alongside innate ones from the olfactory bulb. In other words, a portion of barrel and piriform cortical neurons in mice that express this associative memory receives new synaptic inputs based on their mutual innervations in conjunction with innate synaptic inputs, so that these neurons encode associated new and innate signals, i.e., associative memory cells ^2, 10, 13, 14^. The neurons that encode either one of these signals are called as new memory cells or innate memory cells ^2^. These associative memory cells include glutamatergic neurons, GABAergic neurons and astrocytes ^2, 10, 12, 15^. Moreover, associative memory cells can encode more than two signals ^9, 50, 51^. Paired whisker, odor and tail stimulations lead to odorant-induced and tail-induced whisker motions alongside whisker-induced whisker motion. Associative memory cells in correspondent sensory cortices are recruited to encode three associated signals via their mutual synapse innervations ( 9, 51 and Figure 1). Therefore, associative memory cells and mutual synapse innervations among sensory cortices constitute cellular substrates to memorize specific associated signals, in which the co-activation and simultaneous activity of these cortices are essential for new synaptogenesis and associative memory cell formation. As there are no natural connections among these cortices in mice, this associative learning model can be considered as “artificial” intelligence in animals.

In terms of molecular mechanisms for the recruitment of associative memory cells, some microRNA, such as microRNA-324 and microRNA-133, levels increase ^12, 15^. Associative memory, new synaptic innervations and associative memory cells are attenuated by antagomirs for microRNA-324 and microRNA-133a ^9, 52^. Therefore, microRNA-mediated epigenetic processes, through lowering ttbk1 and tet3 expression, appears to be involved ^9, 51, 52^. The blockade of ttbk1 and tet3 can weaken the limiting factor for the axon prolongation and synapse function ^53, 54^. Therefore, the formation of new synapse innervations and the recruitment of associative memory cells may be based on a chain reaction of intensive spikes, microRNA expression changes and epigenetic-regulated genes and proteins that manage axon prolongation and synapse formation ^9, 10, 52^.

**Figure 2.**
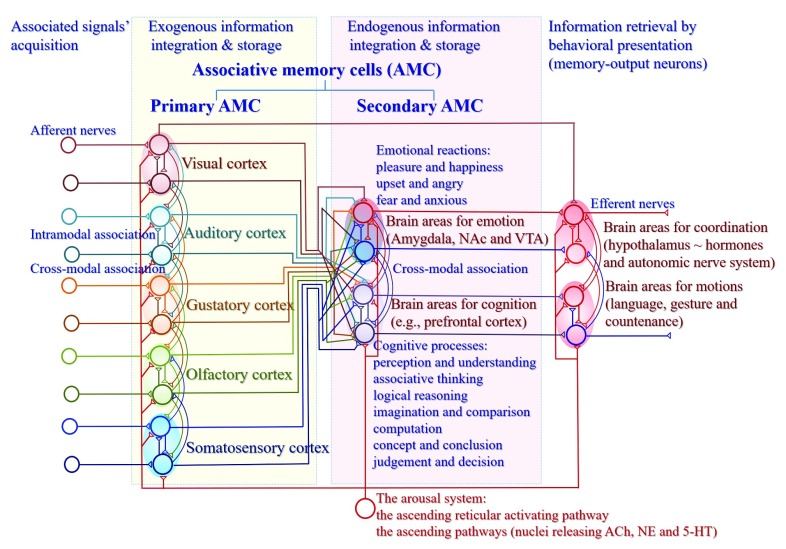
Associative memory cells and their working principle in memory, cognition and emotion. There are four stages of associative learning and memory, associated signals’ acquisition, exogenous information integration and storage, endogenous information integration and storage as well as information retrieval by behavioral presentation. Associative memory cells (AMC) are classified into primary AMCs in sensory cortices that integrate and memorize exogenous information and secondary AMCs in cognition-, emotion- and behavior-related brain areas that memorize endogenous information during associated cognition and emotion events. Cross-modal associative memory cells are recruited by mutual innervations among sensory cortices or between cognition- and emotion-relevant brain regions. Intramodal associative memory cells are recruited by mutual innervations among the neurons in a single-modality sensory cortex, cognition brain area or emotion brain area. In addition to activations by innate input and new mutual innervations from co-activated brain regions to integrate and encode associated signals, associative memory cells are activated by the arousal system including the ascending reticular activating pathway in the brain stem and thalamus as well as the ascending activating pathways from the cholinergic nuclei, midbrain raphe nuclei and locus coeruleus that release acetylcholine (ACh), serotonin (5-HT) and norepinephrine (NE), respectively, which can maintain well wakefulness, permit normal consciousness as well as grant specific alertness and attention. Primary and secondary associative memory cells innervate and activate memory-output cells in the motor cortex for memory presentations by languages, gestures and countenance. Emotional reactions are often accompanied by the activities of autonomic nerves and hypothalamic hormones. Emotion-related brain areas include the amygdala, nucleus accumbens (NAc), ventral tegmental area (VTA) and so on that are involved in emotional reactions. Cognition-related brain areas, such as the prefrontal cortex, work for cognitive processes. The upregulations of AMC number and activity strength can facilitate memory to be impressive, or vice versa. The functional downregulation of motion-related brain areas leads to the inability of memory retrieval and presentation.

**Figure 3.**
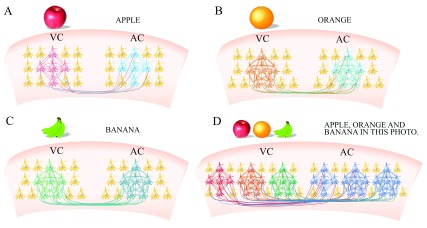
The paradigms for learning and memorizing simple and complicated images and languages. **A**) Learning apple’s image and word “APPLE” composed by letters associatively. Intramodal associative memory cells (red) in the visual cortex (VC) are recruited through their mutual innervations to encode apple image about different features. Intramodal associative memory cells (blue) in auditory cortex (AC) are recruited through their mutual innervations to encode word “APPLE” based on their composed letters by listening the sound of letters and word. The association of apple’s image and APPLE leads to the co-activation of associative memory cells in the visual cortex and auditory cortex, so that mutual synapse innervations between these two groups of associative memory cells as well as cross-modal associative memory cells are recruited. **B**) Learning orange’s image and word “ORANGE” composed with letters associatively. Intramodal associative memory cells (orange) in the visual cortex (VC) are recruited by their mutual innervations to encode orange image about different features. Intramodal associative memory cells (cyan) in auditory cortex (AC) are recruited by through their mutual innervations to encode word “ORANGE” based on their composed letters by listening the sound of letters and word. The association of orange’s image and ORANGE leads to the co-activation of associative memory cells in the visual cortex and auditory cortex, such that mutual synapse innervations between these two groups of associative memory cells as well as cross-modal associative memory cells are recruited. **C**) Learning banana’s image and word “BANANA” composed with letters associatively. Intramodal associative memory cells (green) in the visual cortex (VC) are recruited through their mutual innervations to encode banana image about different features. Intramodal associative memory cells (green) in the auditory cortex (AC) are recruited by their mutual innervations to encode word “BANANA” based on their composed letters by listening the sound of letters and word. The association of orange’s image and BANANA leads to the co-activation of associative memory cells in the visual cortex and auditory cortex, so that mutual synapse innervations between these two groups of associative memory cells as well as cross-modal associative memory cells are recruited. **D**) Associatively learning images (apple, orange and banana) and words “APPLE, ORANGE and BANANA” coactivates associative memory cells in the visual cortex for these images as well as associative memory cells in the auditory cortex for these words. These groups of cross-modal associative memory cells are mutually innervated, and their coactivity upregulate their functional state. These functionally regulated associative memory cells are easily activated by the cues that lead to dominant recall and memory presentation, in which the coactivity-dependent cycle in the refinement and recruitment of associative memory cells is involved. The functionally upregulated cells for encoding images are labeled by dark red, orange and green color, as well as those for encoding words are labeled by dark blue, in comparison with those before their associative coactivation. Cells include ready-recruited neurons and naïve neurons are labeled by yellow. All of the neurons receive their innate input (yellow axons).

Where is the primary location of integration of associated signals? Inhibiting the function of sensory cortices blocks reciprocal cross-modal reflexes ^2, 10^. Applying microRNA antagomirs to these sensory cortices significantly weakens the strength of associative memory as well as the recruitment of new synaptic innervations and neurons that encode associated signals ^9, 52^. Therefore, the primary areas to encode associative memory are likely located in sensory cortices, where mutual synapse innervations and associative memory cells, called as primary associative memory cells, are recruited after associative learning ^3^. It is worth noting that the association of cross-modal sensory signals may occur in visual versus auditory signals, auditory versus olfactory signals, auditory versus taste signals, and auditory versus somatosensory signals, such that primary associative memory cells can be recruited in visual, auditory, olfactory, gustatory and somatosensory cortices through their mutual synapse innervations ( 3 and Figure 2 and Figure 3).

Taking these studies together, we propose the characteristics of associative memory cells in sensory cortices. The co-activation and simultaneous activity of cortical neurons are essential to generate new synaptic innervations and recruiting associative memory cells. They receive new synapse innervations from co-activated sensory cortices for their mutual connections as well as innate sensory inputs. They encode new and innate associated signals for their integrative storage. Their axons project to cognition- emotion- and behavior-related brain regions for cognitive event and memory presentation. The number of associative memory cells and their upregulated refinement influence memory strength and maintenance. The activation of associative memory cells permits logical reasoning, associative thinking, and so on. Their recruitment is influenced by epigenetic-regulated genes and proteins that manage axon prolongation and synapse formation ^9, 10, 52^. In the integration, storage and retrieval of associated signals, the working principle of associative memory cells is based on their reception strength to innate and new synapse inputs, their ability to convert synaptic analogue signals into digital spikes for encoding associated signals and their ability to output sequential spikes ^55– 58^ that will drive behavior-, cognition- and emotion-related brain areas. Thus, synapse innervations to associative memory cells determine the specificity of memory contents. The number, activity level and plasticity of associative memory cells as well as the connection and activity strengths in their input and output partners set up the power and persistence of information storage and memory presentation ^9, 11, 52, 59^. For instance, barrel cortical neurons receive new synapse inputs from the piriform cortex after associative learning in conjunction with innate inputs from the thalamus. Synapse activities induced by odor stimulus will drive barrel cortical neurons toward the threshold of firing spikes under the condition of basal thalamic input, and their spikes then activate motor cortical neurons for odorant-induced whisker motion. With associative memory cells in sensory cortices ^2, 9, 10, 15, 52^, their axon-innervated downstream neurons are able to encode these associated signals ^16, 21, 25, 26, 29– 31^. The stimulation to any of these areas in neural circuits from sensory cortices to behavior- and emotion-related brain nuclei can induce memory presentation ^19, 20, 23, 24, 27, 28^.

## Associative memory cells in cognition-, emotion- and behavior-related brain areas

In addition to primary associative memory cells in sensory cortices for the integration of exogenous signals, secondary associative memory cells that work for the integrative storage of endogenous signals may be recruited in cognition, emotion and behaviors ^3^. This assumption is based on the following facts in life. The content, process, and consequence of logical reasoning and associative thinking can usually be remembered and stated. For instance, we often can tell that images are from previous sights, words from previous reading or listening, tastes from previous eating, and so on. Emotional reactions to various stimulations and operation processes can be recalled for subsequent description. These specific events in the mind may be generated based on the associative storage of previous exogenous signals in sensory cortices, and memorized in brain regions relevant to cognition, emotion or behaviors in an integrative manner. In terms of cellular substrates, the hypothesis is that the association of previously stored associative signals in sensory cortices makes primary associative memory cells strengthening their mutual synapse innervations, convergently innervating downstream neurons and even receiving synapse innervation back. These downstream neurons become to encode associative signals and are recruited as secondary associative memory cells that memorize specific contents in associative thinking and logical reasoning, and their interactions form associative thinking and logical reasoning with the inclusion of sensory origins ^3^.

Certain studies have been conducted to examine the involvement of brain areas including prefrontal cortex, hippocampus and amygdala in memory formation ^24, 60^. Neurons in the medial prefrontal cortex demonstrate a sustained activity after paired stimuli ^25, 26^. Cue-selective neurons are recorded in the inferotemporal cortex after pair association memory ^21^. Neurons in response to conditioned and unconditioned stimuli and their response transformation are detected in the amygdala ^61^. Neurons in the hippocampus and amygdala take part in contextual fear memory ^62^. Memory assemblies for temporal information are overlapped and recorded in the hippocampus ^16^. The activation of engram cells in the amygdala or hippocampus is sufficient to induce fear responses ^19, 20^. This indicates that memory cells are generated in the prefrontal cortex, hippocampus and amygdala for memory retrieval. However, whether these memory cells emerge and are innervated secondarily by associative memory cells in sensory cortices remains to be examined.

In a mouse model of associative learning by pairing whisker and odor signals, neurons that encode whisker and odor signals are detected in the motor cortex and prefrontal cortex ^30, 31^, in addition to barrel and piriform cortices ^2, 12^. Their responses are reduced by inhibiting the activity of barrel or piriform cortical neurons. Their responses and plasticity are sustained in the barrel cortex long-term, and decayed in the motor cortex after pair training ends. Moreover, individual neurons in the prefrontal cortex and motor cortex receive synaptic innervations from barrel and piriform cortices after pairing odor and whisker stimulations ^29– 31^. This provides functional and morphological evidence for the recruitment of secondary associative memory cells in the prefrontal cortex, motor cortex and so on, based on the co-activation of neurons in these areas with primary associative memory cells ( 3 and Figure 2).

Current studies appear to imply that secondary associative memory cells in different brain areas undergo cross-modal connection, similar to primary associative memory cells among sensory cortices ^3^. For instance, the pathway from the ventral hippocampus toward the nucleus accumbens is involved in social memory ^22^. Engram cells in the prefrontal cortex emerge after receiving the inputs from the hippocampus and amygdala during contextual fear memory ^18^. The axon projection from the prefrontal cortex and hippocampus to the amygdala is formed during fear memory ^63^. The pathway from the prefrontal cortex to the striatum plays a crucial role in reward memory ^23^.

Based on these studies, the characteristics of secondary associative memory cells are proposed below. They receive new synapse innervations convergently from primary associative memory cells in those co-activated sensory cortices in cognition and emotion events. They encode endogenous associated signals for their integrative storage in cognition and emotion. The association of cognition process and emotion reaction evokes mutual innervation among secondary associative memory cells in cognition- and emotion-related brain areas. Their axons project to memory-output cells in behavior-related brain areas for memory presentation by language, countenance, gesture and writing. The number of recruited secondary associative memory cells and their upregulated refinement influence the memory strength and maintenance of cognitive and emotional contents. The activation of secondary associative memory cells permits the rehearsal of associative thinking, logical reasoning, emotional reactions and so on. Working principle for secondary associative memory cells is based on their reception of synaptic inputs, their capability to convert synaptic analogue signals into digital spikes for encoding associated signals, as well as their ability to output spikes that drive memory-output cells. Thus, synapse innervations to secondary associative memory cells determine the specificity of memory contents in cognition and emotion. The number, excitability and plasticity of secondary associative memory cells, as well as their connection and activity strengths, set up the persistence and power of information storage and memory presentation. It is pointed out that the outputs of secondary associative memory cells innervate brain areas, such as the hypothalamus and extrapyramidal system, to affect sympathetic/parasympathetic balance, temperature setpoint, food ingestion and hormones to be involved in emotional reactions and behaviors.

Associative memory cells can be recruited among cross-modal sensory cortices or their downstream brain areas related to cognition and emotion through mutual synapse innervations. Moreover, associative memory cells may be recruited based on their mutual innervations in intramodal brain areas, such as associated images to the visual cortex ^64^, associated odors to the olfactory cortex, associated words to the auditory cortex and so on ( Figure 1– Figure 3). Associated signals from a given sensory input to its correspondent sensory cortex may innervate the different groups of neurons. The coactivation of these groups of neurons induces their mutual synapse innervations, so that associative memory cells in a single modality of the sensory cortex are recruited, which memorize intramodal signals with different features, strengths and locations of input signals. These associative memory cells in intramodal sensory cortices fulfill the intramodal memory of these associated signals, such that image one induces image two recall, odor one induces odor two recall and word one induces word two recall, or the other way around ( Figure 1– Figure 3) ^3^. In fact, there is usually a time delay among intramodal signals. The activity persistence in the different groups of neurons in a given sensory cortex influences whether their coactivations overlap to recruit intramodal associative memory cells. The different portions, activity levels and connections of these recruited associative memory cells may determine the storage and retrieval of intramodal signals in different features ^65^. In addition, intramodal associative memory cells may be recruited in brain areas related to cognitive events and emotional reactions ^3^.

## Plasticity in associative memory cells

Cell assemblies formed by the strengthening of neuronal connection due to their correlated activities may be involved in information acquisition, especially the coincidence of activity of presynaptic and postsynaptic cells ^47^. This hypothesis has led neuroscientists to study the involvement of synaptic and neuronal plasticity in memory formation for several decades. Plasticity in synapse connection and neuron activity is presumably the cellular mechanisms underlying learning memory ^5, 6, 66, 67^, such as long-term potentiation and depression in synaptic transmission ^45, 46^ or neuronal activity ^26, 68^. However, some points have been ignored in these studies. Synaptic and neuronal plasticity is examined in brain areas presumably related to memory formation, but not in memory cells. Synaptic plasticity in a given neural pathway does not reveal how multiple signals are integrated and stored in associative memory cells. These uncertainties raise an issue of how these data will be integrated into cellular mechanisms underlying associative memory.

In terms of synaptic plasticity on associative memory cell, the number of excitatory synaptic inputs and the strength of individual synapses on glutamatergic and GABAergic neurons are upregulated, glutamatergic neuron’s outputs are upregulated as well as GABAergic neuron’s outputs are downregulated ^12, 15, 29, 59, 69^. In neuronal plasticity, primary and secondary associative memory cells *in vivo* express activity-dependent upregulation in their active population and activity strength in response to associated signals ^31^, the intrinsic property of glutamatergic associative memory cells is upregulated and the excitability of GABAergic associative memory cells is downregulated ^12, 15, 59, 69^. Mutual innervation among associative memory cells is upregulated ^12, 15^. These factors coordinately shift the balance between excitation and inhibition to more excitation at cortical neurons that may be the driving force in recruiting more mutual synapse innervations as well as glutamatergic and GABAergic associative memory cells, to promote their functional state to an optimal level for storing associated signals, and to facilitate the activation of associative memory cells for the recall and presentation of associated signals ^11^. In associative memory cells with new synapse innervations, their upregulated excitatory input and downregulated inhibitory input can increase their active states to the higher level for receiving and storing new information, i.e., the recruitment of more associative memory cells ^2, 10, 15^. The raised number and function of excitatory synapse inputs strengthen the encoding ability and precision of associative memory cells for information storage and precise and efficient retrieval ^55– 57^. If excitatory associative memory cells are overly active, they activate the neighboring inhibitory neurons to prevent them from the hyperactivity through recurrent negative feedback ^55, 70– 72^.

There are two forms of neuronal excitation plasticity to interpret how neuronal plasticity is involved in the formation and the retrieval of associative memory, i.e., the downregulation of threshold potential to fire spikes and the upregulation of spiking ability to fire more spikes. It has been found that the intensive activity of the neurons by high frequency stimulation, similar to neuronal activation by inputting learnt signals, decreases neuronal threshold potential close to the resting membrane potential, so that the firing of neuronal spikes is facilitated ^68^. Their plasticity in multiple grades ^68^ allows associative memory cells to handle the different groups of associated signals. Furthermore, intensive neuronal activity upregulates cell capacity to fire sequential spikes ^26, 67^. Both mechanisms enhance neuronal capability to encode digital spikes for the recruitment of new synaptic innervations and associative memory cells as well as the retrieval of stored associative signals. These features have been observed in associative memory cells ^12, 15, 59^. These data indicate that the plasticity of neuronal excitability may also play a central role in learning and memory, which is reiterated by a current review ^73^.

Based on the discussion above, we summarize the following points for the integration and storage of associated signals. The formations of primary associative memory cells in sensory cortices and of secondary associative memory cells in cognition/emotion-related brain areas endorse the specificity of the stored associative signals ^2, 3, 9, 10, 12, 15, 52^. The number and functional state of associative memory cells influence the strength and maintenance of information storage as well as the recall and presentation of the stored information ^9– 11, 52^. Structural and functional plasticity at subcellular compartments of associative memory cells determines whether they sensitively integrate associated signals, precisely memorize these signals, and efficiently trigger neurons in their downstream brain areas for memory presentation ^12, 15^. The recruitment of associative memory cells by new synapse innervations and the plasticity of their function states are critical for information storage and retrieval. In addition, both recruitment and refinement of associative memory cells depend upon the simultaneous activity of neurons ^2, 9, 10, 12, 15, 52^. The activities of associative memory cells as a central point comprise coactivity-dependent cycle in their recruitment and refinement, i.e., activity together, wiring together and strengthening together. Highly active neurons in learning associated signals recruit associative memory cells and upregulate their functions. The upregulated population and function state of associative memory cells in these repeated learning events recruit more associative memory cells and upregulate their functions further. This coactivity-dependent positive cycle that is based on functional compatibility between neuronal partners ^58^ can interpret realistic practices under the condition of normal consciousness and high attention, that is, the more learning times is, the more associative memory cell recruitment and refinement is, and the more impressive memory is. It is noteworthy that associative memory cells fall into the active group of neurons in the brain, but non-specific *c-fos*-labelled active cells in the brain may not be memory engrams.

## Working principles of associative memory cells

Associative memory cells are essential for memory formation and related cognitions ^2, 9, 29– 31, 52, 59, 69^. If it is true, their natures and working principle can also be used to interpret the processes of associative learning and memory, such as the efficiency of associative learning, the integrative storage of specifically associated signals, the strength and maintenance of associative memory, the efficiency of memory retrieval, the transformation of simple to complicated information storage, the correlation of associating memory to cognitive process and emotional reactions, and so on. Moreover, memory patterns, such as explicit versus implicit memory, declarative versus non-declarative memory, episodic versus semantic memory as well as transformation between these patterns, remain to be figured out in cellular bases. How associative memory is encoded under the different states of consciousness and attention remains unknown. Here, we discuss the working principles of associative memory cells in these aspects of learning and memory.

The simultaneous activity of neurons in different brain regions is essential for the recruitment of associative memory cells. The co-activation of sensory cortices induces their mutual synapse innervations, such that associative memory cells will be recruited ^2, 9, 12, 15^. The axons of these primary associative memory cells convergently innervate the cognition/emotion-related brain areas to recruit secondary associative memory cells in logical reasoning and associative thinking ^29– 31^. These populations of associative memory cells, based on their received synapse inputs among co-activated brain areas, constitute the memory specificity of associated signals. The coactivity-dependent positive cycle in the recruitment and refinement of associative memory cells promotes the strength and maintenance of associative memory. These results advance a classical hypothesis that the groups of repeatedly co-activated cells become wired and that the strengthening of neuronal wiring forms cell assembles for memory ^47^.

In terms of the driving force to activate and maintain the activities of neurons and associative memory cells, there are three resources, including new synaptic innervations from co-activated brain regions, innate synaptic inputs formed during development, as well as synaptic inputs from non-specific ascending pathways in the arousal system. The ascending reticular activating pathway from the brain stem and the thalamus receives various sensory inputs and widely innervates the cerebral brain to allow wakefulness and consciousness ^74– 76^. The ascending pathways from neuronal axons in the cholinergic nuclei, midbrain raphe nuclei and locus coeruleus innervate the forebrain to maintain alertness and consciousness by releasing acetylcholine, serotonin and norepinephrine ^77– 79^. With this arousal system to uphold the basal activation of associative memory cells, they are able to integrate innate and new synaptic inputs specifically and to memorize these associated signals. Similarly, this arousal system may influence the efficiencies of associative learning and memory retrieval as well as the association of memory with the cognitive process and emotional reaction.

The efficiency of associative learning is affected by the intrinsic property of neurons, their responsiveness to synaptic inputs, and the number of neurons ready to be recruited. Neurons ready to be associative memory cells may have stored signals relevant to the topic that will be learnt, and can be activated by giving topic cues in attention call. The proportion of recruitment-ready neurons influences how the information is acquired and memorized easily as well as how the complicated signals are efficiently learnt (please see below). This is one reason why the efficiency of associative learning is influenced by whether individuals are knowledgeable in the topic to be learnt. In addition, cortical neurons are diverse in their synapse input and intrinsic property ^55^. Neurons with more synaptic inputs and lower threshold potential are easily activated to fire spikes for high learning efficiency ^2, 59^, which triggers a chain reaction of intensive spikes and microRNA expression changes for axon prolongation and synapse innervations ^9, 52^. Thus, activity-dependent upregulation in neuronal excitability and synapse innervations facilitates the learning efficiency.

The efficiency of information recall and memory presentation is affected by the number and functional state of associative memory cells as well as the coactivity-dependent cycle of associative memory cell recruitment and refinement. In general, the recruited number of associative memory cells is proportional to the activated associative memory cells in memory retrieval under the condition of normal consciousness and alertness, so that the efficiency of memory retrieval would be consistent to the efficiency of associative learning ^2, 59^. The functional state of associative memory cells influences how they are easily activated during memory retrieval ^15^. As discussed above, the coactivity-dependent cycle in the recruitment and refinement of associative memory cell will add more associative memory cells into the memory-unit pool in information storage areas, such that the efficiency of memory retrieval would be higher under the condition of normal consciousness and alertness. In addition, whether the stored information can be successfully retrieved is dependent on the functional state of memory-output cells, since the functional downregulation of memory execution cells in the motor cortex leads to the inability of memory retrieval (i.e., memory extinction) though primary associative memory cells are well-maintained in their normal function ^11, 30^.

In the transformation from exogenous signals to endogenous signals and their integrative storages ^3, 29– 31^, the efficiency to correlate associative memory with cognitive processes and emotional reactions is a critical issue. In this process, the interaction between primary and secondary associative memory cells based on their mutual synapse innervation ( Figure 1), as well as the number, function state and plasticity of these associative memory cells, should be accounted during logical reasoning and associative thinking. Thus, cellular processes involved in the efficiency for the learning, storage and retrieval of exogenous associated signals may similarly work for the transformation of exogenous-to-endogenous signals.

Associative learning and memory in life involves a gradual process where individuals memorize associated signals from simple to complication, i.e., the transformation of simple to complicated information storage, in a topic-related manner ^3^. Initially, simple images with different intramodal features and words based on letters are jointly learnt to activate neurons in visual and auditory cortices. With their mutual synapse innervation, associative memory cells (AMC) are recruited in intramodal and cross-modal manners including AMCs for pictures (AMC _PP_), for letters (AMC _LL_) as well as for pictures and words (AMC _PL_). Their plasticity and reactivation will recruit more associative memory cells to initiate the coactivity-dependent cycle in recruitment and refinement, i.e., the first grade of associative memory cells. Through the accumulation of associative memory cells that store simple images and words, they are ready-recruited neurons that become to encode complicated associative signals during learning complicated images and sentences. Subsequently, the complicated images and sentences are associatively learnt to activate the first grade of associative memory cells in visual and auditory cortices. Through their mutual synapse innervation, the second grade of associative memory cells to encode complicated images and sentences are recruited including associative memory cells for complicated pictures (AMC _CPP_), for sentences (organized words, AMC _OWW_) and for pictures and sentences (AMC _PPS_). Through these processes, numerous groups of the first and second grades of associative memory cells are recruited and accumulated in life-time learning. In the advanced learning, multiple grades of associative memory cells are recruited to encode more complicated signals. Once the different groups and grades of associative memory cells are recruited, the subsequent learning and memory will initiate plasticity at these associative memory cells based on their intensive activity ^31^, which become easily activated for quick learning and memory. Reading books or looking at images induces intense activity in certain groups of associative memory cells that encode sentences and images, which leads to activity-dependent upregulation at these associative memory cells. Their low threshold potential to fire spikes ^68^, and active synapse inputs to drive these cells will permit the cues dominantly to activate them for recall of images and sentences, and even the spontaneous activation of these cells to drive secondary associative memory cells for free associative thinking. These associative memory cells will lead to memory presentation by behavior if they successfully drive the activation of memory-output neurons in the motor cortex. It is noteworthy that the complicated signals can also be dissected and memorized through the formation of associative memory cells that are able to encode two signals, three signals, and even more signals ^9^. The partial activations of these associative memory cells lead to the selective recall of these complicated signals.

In terms of the correlation of associative memory cells to memory patterns, such as explicit versus implicit memory, declarative versus non-declarative memory, episodic versus semantic memory and transformation between these patterns, our views are given below. Although these are psychological classifications, there is no clear border line to separate them. Explicit or declarative memory is intentional recall consciously, and implicit or nondeclarative memory is effortless recall without conscious awareness. In fact, the formation of so-called implicit memory has been implicated when individuals initially learn these processes and operations. With long-term practice to be skilled, the recall and expression of these processes and operations no longer require conscious effort. As a result of the coactivity-dependent cycle between the recruitment and refinement of associative memory cells, the repeated activity of primary and secondary associative memory cells will recruit more associative memory cells and upregulate their functions ^2, 12, 15^, as well as strengthen synapse connections from associative memory cells to memory-output cells in the motor cortex ^11, 30^, so that explicit memory can be converted into implicit memory. In other words, there may be a negative relationship between the number and upregulation of associative memory cells and the requirement of consciousness, a homeostasis for memory retrieval. Implicit memory is based on more associative memory cells that are easily activated, supported by phenomena that implicit memory can usually be expressed spontaneously. In explicit memory, episodic memory in individual events can be converted to semantic memory. In general, once associative thinking and logical reasoning with their repetitions place all associative memory cells that store the events with similar topics together to reorganize them into a group of memory cells for general concepts and/or to convergently innervate on another grade of associative memory cells in an abstract manner.

Consciousness is the combinational state of wakefulness and memory, where individuals are aware of and identify themselves and objects in the environment ^80^, which may be based on the basal activation of associative memory cells by the ascending arousal system and the specific activation of associative memory cells by their associated inputs through sensory cues. In this regard, the number and functional state of associative memory cells is proportional to the state of consciousness. The combination of consciousness and a specific alert constitutes attention, i.e., a specific group of associative memory cells are activated. Once individuals are under consciousness, they possess two forms of logical reasoning and associative thinking, i.e., critical versus creative. Critical thinking activates more recruited secondary associative memory cells for evaluation, whereas creative thinking may generate newer secondary associative memory cells for inspiration.

The awareness state can be classified into consciousness and unconsciousness. Sleep can fall into unconsciousness (slow wave sleeping) and incomplete consciousness (fast wave sleeping) ^80^. How do different groups of associative memory cells work together during fast wave sleeping or dreaming? Dreams are often accompanied by high activities in electronic encephalograph and behaviors, such as rapid eye movement, muscle twitch and active respiration/heat beat, indicating high activity in the forebrain. In the meantime, associative memory cells in the large population for specific images and events are presumably activated, especially those cells intensively activated and frequently thought during daytime, such that these images and events are played back. Because of the negative relationship between the number and upregulation of associative memory cells, and the requirement of consciousness, a large portion of associative memory cells can be activated under partial consciousness condition, which makes the playback of images and events being incomplete duplicates of realistic ones. Because playbacks can be recalled and described, associative thinking and logical reasoning (the integration of endogenous signals) based on primary and secondary associative memory cells can be fulfilled under incomplete consciousness ^3^.

The signals from different sensory modalities and various events can be expressed by word-based language during associative thinking and logical reasoning. The associations of these sensations or events with their word descriptions occur during initial learning, where the co-activation of these cortical areas recruits associative memory cells that encode these sensations/events and word descriptions. While the sensations and behaviors are recalled in sequential playbacks, associative memory cells for their word descriptions are triggered, such that word descriptions substitute the complicated images and events to speed up these cognitive processes ^3^. The substitution of words to images is realized based on the recruitment of more associative memory cells and their upregulation in coactivity-dependent cycle manner by repeated practices. However, if words and sensation/events are associated improperly, the correction of these associations is difficult because of the presence of these recruited synapse innervations, associative memory cells and their circuits.

## Modulation of associative memory cells

In addition to specific new synapse innervations and innate inputs, there is no substantial evidence indicating the modulation of associative memory cells by other synaptic inputs and neurotransmitters. The arousal system including the ascending reticular activating pathway ^74, 75^ and ascending activating pathways from the neuronal axons of the locus coeruleus, midbrain raphe nuclei and cholinergic nuclei ^77– 79, 81^ widely innervates the forebrain to maintain wakefulness and to permit consciousness. Their released neurotransmitters, acetylcholine, serotonin and norepinephrine, theoretically modulate the functional states of ready-recruited neurons and associative memory cells. The activity of this arousal system maintains the basal activation of associative memory cells that integrate new and innate synaptic inputs, as well as memorize these associated signals specifically. In other words, the alert and reward may facilitate the recruitment and refinement of associative memory cells.

The modulation of learning memory by acetylcholine, norepinephrine and serotonin has been documented ^82– 85^. The direct activation of acetylcholine M1-type receptors on hippocampal interneurons contributes to learning memory ^86^. The infusion of norepinephrine or adrenoceptor agonist into the amygdala or the prefrontal cortex enhances memory formation, which coordinates with the action of stress hormone ^87^. The augmented and reduced activities of serotonin neurons lead to bidirectional influence on memory and cognition ^88, 89^. The action of dopamine onto type-I and type-V receptors in the forebrain and the hippocampus plays an important role in spatial learning and memory ^90, 91^. These data provide the evidence for neurotransmitters, such as acetylcholine, serotonin, norepinephrine and dopamine, as well as stress hormone to modulate learning and memory. How these neurotransmitters act onto the presynaptic inputs of associative memory cells to influence transmitter release or the bodies of associative memory cells to influence their excitability remains to be addressed.

It has been found that serotonin facilitates the neuron excitability and neuron responses to synaptic inputs ^92, 93^, and that the activation of dopaminergic neurons facilitates synaptic bouton formation and postsynaptic neuron activity in their target regions ^94^. The plasticity of these monoaminergic neurons may modulate the recruitment and refinement of associative memory cells, and in turn influence memory formation and memory-related cognitions. This modulation supports the fact that the high levels of wakefulness, consciousness, attention and motivation based on the active monoaminergic and cholinergic neurons elevate the efficiencies of associative learning and memory retrieval.

## The impact of associative memory cells on physiology and pathology

The memory of associated signals is important for establishing bidirectional alertness and prediction in life. With associative memory cells based on primary and secondary locations, as well as grade one, two or more integrations, one signal induces the recall of its associated signals and the expression of their respective behavior, or the other way around, such that individuals are able to fulfill logical reasoning and associative thinking, as well as to predict future events in a forward and backward manner. Moreover, associative memory cells in each of co-activated brain areas encode the associated innate signal and newly learnt signal. Each of associated signals is memorized at multiple brain areas, which largely reduces the chance of memory loss ^2, 3^. The storage of multiple signals in an associative memory cell strengthens the efficiency of memory retrieval ^9^. The storage of multiple signals in a cortical area and the recall of one signal triggered by multiple signals enable these individuals to strengthen their abilities in memory retrieval and well-organized cognitions ^3^.

It is widely accepted that the normal consciousness and attentiveness are important for memory formation ^48, 95, 96^, which can be explained by associative memory cells and their characteristics. With the arousal system to maintain wakefulness and the activation of recruitment-ready neurons by giving topic cue in attention call, their activation and activity allow them to encode associated signals. These recruited associative memory cells under the wakefulness condition allow for individuals to identify themselves and objects in their environment, which constitutes consciousness. The consciousness supports the activation and activity of associative memory cells to enter the activity-dependent positive cycle in their refinement and recruitment, such that more associative memory cells are recruited and impressive memory is generated in mind.

The age-related change in the efficiency of learning and memory throughout life is a well-known phenomenon ^97, 98^. There is an uprising-peak-decline process of associative learning and memory ^2^. In terms of cellular mechanisms, synaptic potentiation becomes matured during the postnatal development ^99^, and neuronal excitability in mouse cortical neurons is upregulated to a plateau level at postnatal day 22 ^68^, which matches well with dynamic changes in associative memory ^2^. The plasticity of neurons and synapses with the recruitment of associative memory cells in postnatal development initiates the coactivity-dependent cycle to recruit and refine associative memory cells, so that more associative memory cells are recruited to increase the efficiency of learning and memory ^59, 69^. In aged mammalians, the accumulations of β-amyloid and phosphorylated tau-protein in the brain may influence axon prolongation and synapse formation ^9, 52^ to block the recruitment of associative memory cells and/or to impair those recruited associative memory cells for memory deficits.

In terms of memory maintenance and extinction, the recruitment and plasticity of associative memory cells are not significantly decreased, but the activity of memory-output neurons in the motor cortex is lowered ^11, 30^. The sustained presence of associative memory cells as well as the recruitment of more associative memory cells during brain excitation confer associative memory to be recalled in lifetime, in which the information can be retrieved as long as their innervation onto memory-output neurons successfully drive them to be functionally active. It is noteworthy that memory recall shows different patterns in spontaneous, cue-induced and realistic object-triggered manner with the age. For instance, spontaneous recall occurs in child age and/or brain excitation, cue-induced recall usually occurs in the young and adults, and real object-triggered recall occurs in senior individuals. In addition, when the brain is highly excited in many regions, such as euphoria perception, extreme fear and strong stimulus, more associative memory cells are recruited in these areas through their mutual innervations, so that impressive memory and spontaneous recall to these experiences are generated in individual lifetime ^11, 30^. It is difficult to remove newly formed synapse innervations and recruited associative memory cells for the relief of fear memory. Alternative ways are the avoidance of fear stimulation and the induction of happiness to rebalance these two states to weaken fear memory, since the lack of uses in neural circuits related to fear memory ^3^, especially from associative memory cells to memory-output neurons, may drive them to be functionally silent. In the brains of individuals with a history of substance abuse or addiction, primary and secondary associative memory cells related to these events are recruited in large amounts and in extensive areas under euphoria conditioning, leading to potential relapses in their lifetime ^3^. Strategies for these individuals may include the avoidance of the environmental cues associated with substance abuse to weaken the output of the relevant associative memory cells, as well as the establishment of alternative sources of happiness to recruit associative memory cells, such that the rebalance of these two states will strengthen the memory-output pathway for happiness.

The proper upregulation of active neurons leads to their recruitment of associative memory cells, and the upregulation of associative memory cells facilitates the joint storage of associated signals ^3, 15^. These processes initiate the coactivity-dependent cycle of memory cell recruitment and refinement, so that more associative memory cells are recruited. However, the further upregulation of associative memory cells, e.g., the dysfunction of GABAergic neurons in schizophrenia and epilepsy ^100, 101^, allows associative memory cells to be overlaid and widely activated. The upregulated associative memory cells in sensory cortices will lead to hallucination, and the upregulated associative memory cells in cognition and emotion-related brain areas leads to delusion.

## Conclusions

Associative memory cells are the basic unit to encode associated signals in objects and environments. Their recruitment and functional upregulation essentially determine the efficiency of learning and memorizing specifically associated signals. Their activation and persistent activity lead to the recall of memorized associated signals in the mind, and the presentation of stored signals by behaviors if they successfully activate memory-output cells. Morphological basis for these associative memory cells to encode associated signals is their receptions of innate input and new synapse innervations from co-activated brain areas. Based on the localization of associative memory cells, they are classified into primary groups to integrate exogenous signals in sensory cortices and to innervate neurons in cognition and emotion brain areas, as well as secondary groups to receive innervations from primary groups and to integrate endogenous signals during cognitive processes. Based on the complication of integrating associated signals, associative memory cells are classified into grade one, grade two and so on, whose activity-dependent upregulation works for the storage and retrieval of complicated signals. Associative memory cells with their upregulation lead to them being more active and recruit more ready-neurons to be associative memory cells, i.e., a coactivity-dependent cycle in the recruitment and refinement of associative memory cells for impressive memory in repetitive learnings. Associative memory cells are the basic unit for the storage of associated signals that influence the contents of cognitions and emotion reactions. The consequences and processes of cognition and emotion recruit more associative memory cells for them to be stored. The cycles in memory and cognition allow the experiences, capabilities and skills to be strengthened. In addition, the functional state of associative memory cells is modulated by the arousal system from the brain stem including the ascending reticular activating pathway and the ascending pathways from midbrain raphe nuclei, locus coeruleus, cholinergic nuclei and substantial nigra, which affect the efficiency of learning and memory. Working maps for associative memory cells are given in Figure 1– Figure 3.
