# Supplementary material for: Associative memory cells and their working principle in the brain
Source: F1000Res. 2018 Jan 25;7:108. [Version 1] doi: 10.12688/f1000research.13665.1 (PMC5806053; doi:10.12688/f1000research.13665.1)
Supplement: Peer review history

# Peer review: Referee response for version 1

**DOI:** 10.5256/f1000research.14845.r31454

**Published:** 2018-03-05

**Authors:** Jie Wu

**Affiliations:** 1 Department of Neurobiology, Barrow Neurological Institute, St. Joseph's Hospital and Medical Center , Yunfu, Phoenix, AZ, USA

Associative memory cell is a new term to describe the special groups of brain neurons that play the critical role in information/signal acquisition, integration and storage of exogenous associated signals. In this review Dr. Wang has provided a comprehensive picture of these cells, their working principle and modulation. I think this article provides new insights into improved understanding of molecular/cellular mechanisms of cognition, emotion and behaviors. The manuscript is well written and the figures are nicely presented and make sense. I recommend publishing this paper in this journal.

 Following are some my questions and hopefully authors can appropriately address them in this article.

  

1. What are the basic phenotypes of the “associative memory cells”, are they glutamatergic, GABAergic cholinergic, or others?
2. Do these “associative memory cells” play the unit function such as acquisition, integration and storage of exogenous associated signals?
3. Have these “associative memory cells” a special distribution in the brain?
4. Do these “associative memory cells” form the specific circuits that are closely associated to the cognition and emotion?
5. What changes of these “associative memory cells” in the diseases that exhibit cognitive deficits including Alzheimer’s disease, drug addiction, schizophrenia.

I have read this submission. I believe that I have an appropriate level of expertise to confirm that it is of an acceptable scientific standard.

# Peer review: Referee response for version 1

**DOI:** 10.5256/f1000research.14845.r30315

**Published:** 2018-02-08

**Authors:** Wen-Jun Gao, Bo Xing, Yu-Xiang Zhang

**Affiliations:** 1 Department of Neurobiology and Anatomy, Drexel University College of Medicine, Philadelphia, PA, USA

In the present review, Wang and Cui have summarized their previous works about associative memory cells and made a hypothesis of how it works. Specifically, they named the cells that process exogenous associated signals as associative memory cells which could be classified as primary (located in sensory cortices) and secondary (located in cognition-, emotion-related brain areas). The authors also discussed the molecular changes and plasticity of the associative memory cells and raised an interesting hypothesis about working principles of these cells. Overall, this review is compelling, well-written, and timely needed for the field. However, there are some points that are not clear:

1. The authors raised several interesting questions in the manuscript. But it would be helpful for the authors to provide a perspective section to elucidate how these questions should be addressed at the end of the manuscript.  
2. Figure 1 and the related paragraph. The authors’ claim that mutual innervation is the major pattern of primary and secondary associative memory cells connection. This model is quite simple and easy to understand. However, are there any other patterns exist in the associative memory cells? For example, the neocortex is hypothesized to function as cortical column. Does the column model fit in the associative memory cells connection as well?
3. The authors described the functional plasticity in associative memory cells. Do these cells also exhibit morphological changes? For example, what about the spine density or spine shape changes during the memory acquisition or storage?
4. Page 3, In terms of molecular mechanisms, there are many molecular changes during the process. It is not clear why the authors point out microRNA, which is non-coding RNA.
5. Page 6, the authors stated that individual neurons in the prefrontal cortex and motor cortex receive synaptic innervations from barrel cortices……This might be the case for motor cortex. To our knowledge, prefrontal cortex does not receive innervations from the barrel cortex directly.

Page 6, reference 26 is not related to the prefrontal cortex.

We have read this submission. We believe that we have an appropriate level of expertise to confirm that it is of an acceptable scientific standard.

# Peer review: Referee response for version 1

**DOI:** 10.5256/f1000research.14845.r30314

**Published:** 2018-02-05

**Authors:** Ru-Rong Ji

**Affiliations:** 1 Department of Anesthesiology, Duke University Medical Center, Durham, NC, USA

This is a timely review on an important topic, associative memory. The first author is a leader in this area. All the figures are well presented. Figure 1 is very informative. I only have a few minor concerns.

 It will help if the authors can include a table to summarize key literature related to this topic.

 Are there any differences in cellular signaling mechanisms between primary and associated memory? If so, a table to summarize similarities and differences will be very useful.

 Do glial cells play a role in associative memory?

 No pain, no gain? Is pain also associated with this type of memory?

I have read this submission. I believe that I have an appropriate level of expertise to confirm that it is of an acceptable scientific standard.
